# The Optimal Timing for Using Capsule Endoscopy for Patients with Gastrointestinal Bleeding

**DOI:** 10.1155/2021/7605324

**Published:** 2021-03-27

**Authors:** Chao-Chin Chao, Lein-Ray Mo, Susan C. Hu

**Affiliations:** ^1^Department of Gastroenterology, Kaohsiung Municipal Gangshan Hospital, Kaohsiung City 820, Taiwan; ^2^Department of Gastroenterology, Tainan Municipal Hospital, Tainan City 701, Taiwan; ^3^Department of Public Health, College of Medicine, National Cheng Kung University, Tainan City 701, Taiwan

## Abstract

**Objectives:**

Capsule endoscopy (CE) is a useful diagnostic modality for patients with occult gastrointestinal (GI) bleeding. However, most previous studies utilizing CE have focused on techniques, patient characteristics, safety and feasibility, and case analyses. Studies evaluating the optimal timing for utilizing CE, which is an essential factor for obtaining a better diagnostic yield, remain scarce in the literature. Considering that a CE examination is expensive, we, therefore, undertook this study to evaluate, analyze, and determine the optimal time for performing CE in patients with occult GI bleeding.

**Methods:**

Seventy-five patients were initially recruited, but finally, sixty patients with significant GI bleeding with an unknown etiology after traditional endoscopic examinations were included in the study. All data were collected from a local hospital in Taiwan, encompassing the period from 2010 to 2018. The relationship between the timing of CE examination and the diagnostic correction rate (DCR) was then analyzed statistically.

**Results:**

More female (58.3%) and older adult (68.3%) patients were in our study. Based on the four analytical models used in the study, the results showed that the most optimal time to perform CE is within three days after GI bleeding occurs.

## 1. Introduction

In approximately 5% of patients with gastrointestinal (GI) bleeding, the cause remains elusive and difficult to identify, even after using traditional conventional means of identification [1, 2]. After endoscopic procedures in both the upper and lower GI tract, the bleeding location can usually be identified in about 50% of the cases mentioned above. However, the remaining half of those patients whose bleeding source remains unidentified or lacks confirmation using traditional means can be referred for capsule endoscopy (CE) when the small bowel is suspected to be the culprit [3, 4]. Therefore, CE is extremely valuable, is less invasive than push enteroscopy, and plays a vital role in treating these patients [5–8].

Although CE is an adjunct to traditional endoscopy and provides a more precise diagnosis in patients with an elusive source of bleeding, the cost of the procedure is relatively high. Thus, determining the optimal timing for its utilization is critical in order to obtain the maximum impact [9, 10]. In patients with GI bleeding, it is common practice to perform an endoscopy to determine the source. If the bleeder is not identified on the first try, the procedure is repeated. However, if the second attempt fails, CE is usually requested. As a result, much time is wasted, and doctors may have already missed the opportune time for CE examination to identify the bleeding source [11].

CE has been in use for more than two decades. However, most studies in the literature focus on the improvement and refinement of the technique itself [10–13]. Studies analyzing the optimal timing for its utilization remain sparse [9, 14–18]. We, therefore, undertook this study in order to address this issue. Specifically, we aimed to examine the relationship between the timing of CE and the diagnostic correction rate (DCR) in patients with GI bleeding.

## 2. Materials and Methods

The Materials and Methods contains sufficient details so that all procedures can be repeated. It is divided into headed subsections for the methods described.

### 2.1. Patients

All patients were recruited from a local hospital in Kaohsiung City, Taiwan. Patients were included in the study when they had a passage of tarry or bloody stools with an obscure etiology after both esophago-duodenoscopic and colonoscopic examinations. Patients were excluded when they were less than 16 years old, unable to swallow, suspected of small bowel obstruction, or had an implanted permanent cardiac pacemaker. Ultimately, 75 consecutive patients were included in the study population from January 2010 to December 2018. Fifteen patients were later excluded after they exhibited non-GI bleeding.

### 2.2. Procedures for the CE Examination

Briefly, the patients were required to take a cathartic fleet bottle before the examination at exactly 8 : 00 pm. Then, they fast until 9 : 00 am the next day, when the procedure began. The protocol was as follows:

- An assistant helped the patient put on the equipment
- Then, the battery life and signal transmission were verified for any problems
- The patient then swallowed the CE
- The patient was allowed to drink water only after 2 hours and could not ingest any form of food until approximately 4 hours later
- The assistant was allowed to remove the recording device after 10 hours
- The doctor then downloaded the recoded data, analyzed it, and made a final diagnosis

## 3. Analytical Models and Hypotheses

There were four analytical models used in this study. The first was the traditional method, or Model 1, which was used to calculate the DCR based on the various days during which GI bleeding occurred. Then, Models 2-4 (the dichotomous models) were used to compare DCR results by combining the different times when CE was performed relative to the first incidence of GI bleeding. The four corresponding hypotheses are as follows:

*H01*: there were no differences in the DCR after performing CE across various days after the appearance of GI bleeding

*H02*: there were no differences in the DCR after performing CE between 1 and more than 1 (≥1) day after the first bleeding episode

*H03*: there were no differences in the DCR after performing CE between 1-2 and more than 2 (≥2) days after the first bleeding episode

*H04*: there were no differences in the DCR after performing CE between 1-3 and more than 3 (≥3) days after the first bleeding episode

## 4. Statistical Methods

In the analysis, continuous variables are presented as mean and standard deviation (SD) for normally distributed variables. Categorical variables are shown as frequencies and percentages. A Pearson's chi-square test was used for the evaluation of categorical variables. Two analytical approaches, including a traditional analysis and a dichotomous analysis, were implemented to obtain the association between the CE examination timing and the DCR for GI bleeding. All data analyses were conducted using SPSS 12.0 (SPSS Inc. Chicago, IL, USA), where an *α* value of equal (=) to 0.05 was considered significant.

## 5. Results

### 5.1. Patient Characteristics and CE Examination Outcomes

A total of 60 patients were included in the final analysis. Twenty-five (41.7%) were male, and 35 (58.3%) were female. Nineteen (31.7%) were <65 years old, and 41 (68.3%) were ≥65 years old. Forty (66.7%) had small bowel bleeding, which was marked or labeled as “Abnormal,” and 20 (33.3%) patients who did not have small bowel bleeding were marked as “Normal.” Fifty-four patients presented with melena, while the rest had hematochezia.

**Figure 1:**
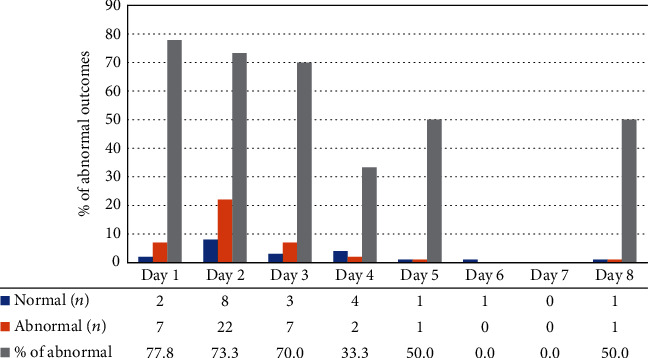
Outcome of capsule endoscopy examination.

Figure 1 shows the outcomes of the CE examination performed on different days for various time periods after the first episode of GI bleeding. Nine (15%) patients had their CE examination performed on the first day; 30 (50%) had it on the second day; 10 (16.7%) had it on the third day, and 11 (18.3%) patients underwent CE on the fourth day and beyond. A definitive diagnosis or correct identification of the bleeding source was obtained in 77.6, 73.2, 70.0, and 36.4% of the cases, respectively.

### 5.2. Hypothesis Testing for the Optimal Timing of the CE Examination

The results of the four hypotheses tested in this study are shown in Table 1. In the case of the traditional approach, there were no significant differences across the different days during which the CE examination was administered after the onset of bleeding (Model 1). For the dichotomous approach, the use of CE on the first day (9 patients) was compared with 51 patients whose examinations were performed on the second day of bleeding (Model 2). After the analysis, there was no statistical difference noted in the DCR (*p* = 0.704). In Model 3, where the CE performed within the first two days of bleeding (39 patients) was compared with that performed on or beyond three days after bleeding (21 patients), there was also no significance noted (*p* = 0.096). However, in Model 4, when the CE has been done within three days (49 patients) compared with the CE performed on or beyond four days after bleeding (11 patients), there was a significant difference in the DCR (*p* = 0.031).

### 5.3. Gender and Age Differences in the CE Examinations

In this study, we also investigated whether gender and/or age influenced the DCR for CE examinations on patients with GI bleeding.  Table 2 shows that 80% of the female patients had small bowel bleeding compared with 48% of their male counterparts. This difference, where 32% more female patients had a greater likelihood of having small bowel etiology as their bleeding source, significantly influenced the DCR (*p* = 0.013) and suggested that gender could be a factor. However, age did not significantly affect the DCR (*p* = 0.146).

In Table 3, it can be seen that more than 90% of the female patients were identified with “Abnormal” outcomes within three days after GI bleeding, versus only 68% of the males within the same inclusion date. Statistically, this proved that the timing of CE examinations also significantly impacted the DCR based on gender (*p* = 0.039).

### 5.4. Findings of the CE Examinations

Based on the clinical characteristics of 40 patients with “Abnormal” CE results, as shown in Table 4, the majority (67.5%) of the patients had small bowel bleeding without a definite or confirmed underlying etiology. This was because the culprit lesions were covered with blood and blood clots when the CE passed the area, hindering clear, complete visualization of the cause of bleeding. Tumors were identified in 10% of the cases, and angioectasia was noted in 7.5%.

## 6. Discussion

Three important caveats should be discussed based on the results of this study. Firstly, the optimal time for CE examination is within three days after the occurrence of GI bleeding, as the DCR on the first to third day reached 77.8, 73.3, and 70%, respectively. However, the DCR fell to approximately 36.4% when CE was performed on the fourth day or later. Based on our analysis, the preferred time for CE examination is on the third day or within three days after the first episode of bleeding. However, no statistically significant differences were noted when using the traditional approach to analyze these four treatment groups.

Secondly, when using the dichotomous approach in analyzing our data, wherein the different time periods were compared with each other, Models 2 and 3 also did not show any significant differences. However, in Model 4, the DCR reached a value of 73.5% when the CE was done within three days of bleeding, with a significant decrease in the DCR when performed on or beyond four days, significantly reducing its diagnostic impact.

Lastly, in terms of significant gender differences, our results showed significant differences between the DCR of male versus female patients. This may be explained by the fact that more female patients (91.4%) underwent CE examinations within the first three days instead of only 17 (68%) male patients. We do not have a good explanation as to why this was the case, except for possibly the small sample size. However, one cannot deny that “optimal timing” remains the most critical factor for improving the DCR in a CE examination.

Although current guidelines by the European Society of Gastrointestinal Endoscopy (ESGE) recommend performing CE examinations within 14 days after a bleeding event [19], our results suggest that the best time to maximize the diagnostic yield of CE is within three days of bleeding, which concurs with the findings of a few other studies in the literature [14, 18].

In clinical practice, regular endoscopic examinations are usually performed once or twice to identify the exact GI bleeding source. Only if bleeding persists without a definitive identifiable source will CE examinations be requested. In this study, we proved that diagnostic effectiveness and impact are highly correlated with the time the CE is performed concerning the initial onset of bleeding. Forty out of the 60 (67%) patients with “Abnormal” results had confirmed diagnoses or complete identification of the bleeding source. These included 27 cases of small bowel bleeding, 4 angioectasias, 4 tumors, and 2 cases of ulcer bleeding. These etiologies are similar to those found in other studies [9, 14, 16], and accordingly, it can be inferred that the timing of the CE examination could lead to earlier diagnosis and more effective therapy [20].

One of the dilemmas associated with CE examinations is to diagnose small bowel bleeding without a clear-cut underlying etiology [3, 11]. In this study, approximately 55.6% and 77.3% of the “Abnormal” cases were diagnosed as small bowel bleeding without a definitive underlying etiology, as explained earlier, on the first and second days of the procedure, respectively. For the patients who underwent CE on the third day of bleeding, 71.4% of patients had the bleeding source correctly identified, which was statistically significant. This means that early utilization of CE, “the earlier, the better” mantra, may not hold in these cases and may not lead to a definitive or accurate diagnosis. More blinded and randomized studies with a larger sample size are warranted to address these issues.

There are several limitations in this study. First, this is a retrospective study in a single hospital, which is the most disadvantage of our manuscript. However, the enrollment and treatment protocols were standardized, potentially reducing the drawbacks of the study design. Second, the small sample size could also have affected the reliability of our study. However, some previous studies with a small sample size of fewer than 100 patients still yield good results [17, 21, 22]. More prospective studies with a larger population or using multicenter are warranted to confirm the findings better. With the maturity of the CE procedure and advanced device availability, we suggest further studies are needed to address the optimal timing for performing CE to obtain the maximal diagnostic yield and impact and to render earlier and more precise treatment options in these patients.

## 7. Conclusions

It was concluded that the optimal timing for CE examination is within three days after the onset of GI bleeding in the study. If the GI bleeding has occurred for more than three days with no change in the patient's clinical condition or severity, CE can be deferred and performed immediately the next time bleeding occurs. Gender was found to have a significant influence on the DCR in this study but that may have been due to the fact that more female patients had their CE performed within the first three days.

## Figures and Tables

**Table 1:** Results of CE examinations using the traditional analysis.

	Normal	Abnormal	*X* ^2^	*p* value
	*N* (%)	*N* (%)		
Model 1			5.70	0.127
Day 1	2 (22.2)	7 (77.8)		
Day 2	8 (26.7)	22 (73.3)		
Day 3	3 (30.0)	7 (70.0)		
≥4 days	7 (63.6)	4 (36.4)		
Model 2			0.59	0.704
Within 1 day	2 (22.2)	7 (77.8)		
≥2 days	18 (35.3)	33 (64.7)		
Model 3			2.97	0.096
Within 2 days	10 (25.6)	29 (74.4)		
≥3 days	10 (47.6)	11 (52.4)		
Model 4			5.57	0.031^∗^
Within 3 days	13 (26.5)	36 (73.5)		
≥4 days	7 (63.6)	4 (36.4)		

^∗^Significant, *p* < .05.

**Table 2:** Diagnostic correction rates (DCR) by gender and age.

	Normal	Abnormal	*X* ^2^	*p* value
	*N* (%)	*N* (%)		
Gender			6.72	0.013^∗^
Male	13 (52.0)	12 (48.0)		
Female	7 (20.0)	28 (80.0)		
Age			2.47	0.146
<65	9 (47.4)	10 (52.6)		
≥65	11 (26.8)	30 (73.2)		

^∗^Significant, *p* < .05.

**Table 3:** The optimal timing for CE examination by gender.

GI bleeding days	Male	Female	*X* ^2^	*p* value
	*N* (%)	*N* (%)		
Within 3 days	17 (68.0)	32 (91.4)	5.35	0.039^∗^
≧4 days	8 (32.0)	3 (9.4)		

^∗^Significant, *p* < .05.

**Table 4:** Findings of abnormal examinations from the capsule endoscopy (*n* = 40).

Clinical diagnosis	1-3 days	≧4 days	Total
	*N* (%)	*N* (%)	*N* (%)
Small intestinal bleeding (without underlying etiology)	24 (88.9)	3 (11.1)	27 (67.5)
Tumor	4 (100.0)	0	4 (10.0)
Angioectasia	4 (100.0)	0	4 (10.0)
Ulcers	3 (100.0)	0	3 (7.5)
Others	1 (50.0)	1 (50.0)	2 (5.0)

## Data Availability

The data used in this study are restricted by the Institutional Review Board of Show Chwan Memorial Hospital (IRB1090502) to protect patient privacy. Data are available from Chao-Chin Chao (chawpp@mail2000.com.tw) for researchers who meet the criteria to access confidential data.
